# Cholinergic modulation of spatial learning, memory and navigation

**DOI:** 10.1111/ejn.14089

**Published:** 2018-08-19

**Authors:** Nicola Solari, Balázs Hangya

**Affiliations:** ^1^ Lendület Laboratory of Systems Neuroscience Department of Cellular and Network Neurobiology Institute of Experimental Medicine Hungarian Academy of Sciences Budapest Hungary

**Keywords:** acetylcholine, basal forebrain, hippocampus, posterior parietal cortex, retrosplenial cortex

## Abstract

Spatial learning, including encoding and retrieval of spatial memories as well as holding spatial information in working memory generally serving navigation under a broad range of circumstances, relies on a network of structures. While central to this network are medial temporal lobe structures with a widely appreciated crucial function of the hippocampus, neocortical areas such as the posterior parietal cortex and the retrosplenial cortex also play essential roles. Since the hippocampus receives its main subcortical input from the medial septum of the basal forebrain (BF) cholinergic system, it is not surprising that the potential role of the septo‐hippocampal pathway in spatial navigation has been investigated in many studies. Much less is known of the involvement in spatial cognition of the parallel projection system linking the posterior BF with neocortical areas. Here we review the current state of the art of the division of labour within this complex ‘navigation system’, with special focus on how subcortical cholinergic inputs may regulate various aspects of spatial learning, memory and navigation.

**Abbreviations**
ACh: acetylcholine
BF: basal forebrain
BFCN: basal forebrain cholinergic neurons
GPi: globus pallidus internus
HD: head‐direction cell
MCI: mild cognitive impairment
MCPO: magnocellular preoptic nucleus
MS: medial septum
MWM: Morris water maze
NBM: nucleus basalis magnocellularis
PPC: posterior parietal cortex
PTD: pyrithiamine‐induced thiamine deficiency
RAM: radial arm maze
RSC: retrosplenial cortex
SI/EA: substantia innominata/extended amygdala
VP: ventral pallidum
VR: virtual reality

## 1 INTRODUCTION

When faced with novelty, most mammals increase their exploratory behavior to facilitate establishing and refining the internal representation of the novel element that could be for instance a change in the surroundings, a novel object in familiar settings or a conspecific. As a special case, animals engage in active exploration when placed in a novel environment to gain spatial knowledge and represent the physical landscape. In the past century, after a series of seminal experiments on rats navigating in a complex maze, Edward Tolman put forward the proposition that animals, including humans, can acquire large numbers of sensory cues and use them to build a mental image of the environment. With this internal representation of physical space they can navigate to a goal by knowing where it is embedded in a complex set of environmental features (Tolman, 1948; Tolman & Honzik, 1930a,1930b). This idea is known today as the cognitive map, an abstract ensemble of environmental relationships and paths which determines the possible routes of actions. Although generally discussed in the context of physical space, the concept of the cognitive map can be generalised to representations of abstract spaces as well, such as hierarchy or time‐series (Epstein, Patai, Julian, & Spiers, 2017).

Navigation can be defined as the process of determining and maintaining a course or trajectory from one place to another (Gallistel, 1990). In cognitive neuroscience navigation is regarded as a complex goal‐directed behavior that involves processing a variety of sensory and proprioceptive stimuli, storage and recall of information and elaboration of plans. There are many ways of finding a goal location, from simple direct approach to an easy‐to‐locate proximal target (local navigation) to using well‐learned routes and cognitive maps (wayfinding). In the simplest case (target approaching or taxis), the goal is directly detectable and the agent only needs to orient towards the observable goal or landmark nearby and then maintain this direction (beacon, O'Keefe & Nadel, 1978). In more complex situations when the goal is not visible, it might still be possible to locate it using visible distal cues, which can then be used to compute the goal direction (guidance, Morris, 1981). To reach a destination outside the local environment, the subject can recongise and approach sequential landmarks or proximal places step‐by‐step (recognition‐triggered response or direction, Mallot & Gillner, 2000) or concatenate multiple recognition‐triggered responses in a route (route following or topological navigation). Notably, a route is a rigid construct that do not involve the creation of a new path. The latter is only possible using a map‐based strategy, which capitalises on the relational organization that is characteristic to the cognitive map.

The wanderer has access to two kinds of cues: those generated by proprioception including vestibular feedback (idiothetic) and those emerging from the environment such as landmarks (allothetic). Similarly, spatial information can be represented both by subject‐based coordinates in a self‐centered (egocentric) frame or by world‐based coordinates in a world‐centered (allocentric) frame (Committeri et al., 2004; Galati, Pelle, Berthoz, & Committeri, 2010; Klatzky, 1998, but see also Meilinger & Vosgerau, 2010; Filimon, 2015). In natural conditions, idiothetic and allothetic signals can be combined to optimise navigation (Poucet et al., 2013; Sjolund, Kelly, & McNamara, 2018), but may be weighed differentially based on their perceived reliability (Chen, McNamara, Kelly, & Wolbers, 2017). Thus, the selection of appropriate navigational strategies is primarily determined by the perception of space, that is, by the nature of the cues that can be used for navigation, modified by the subjects’ individual predispositions and expectations (Ishikawa & Montello, 2006; McIntyre, Marriott, & Gold, 2003).

**Figure 1:**
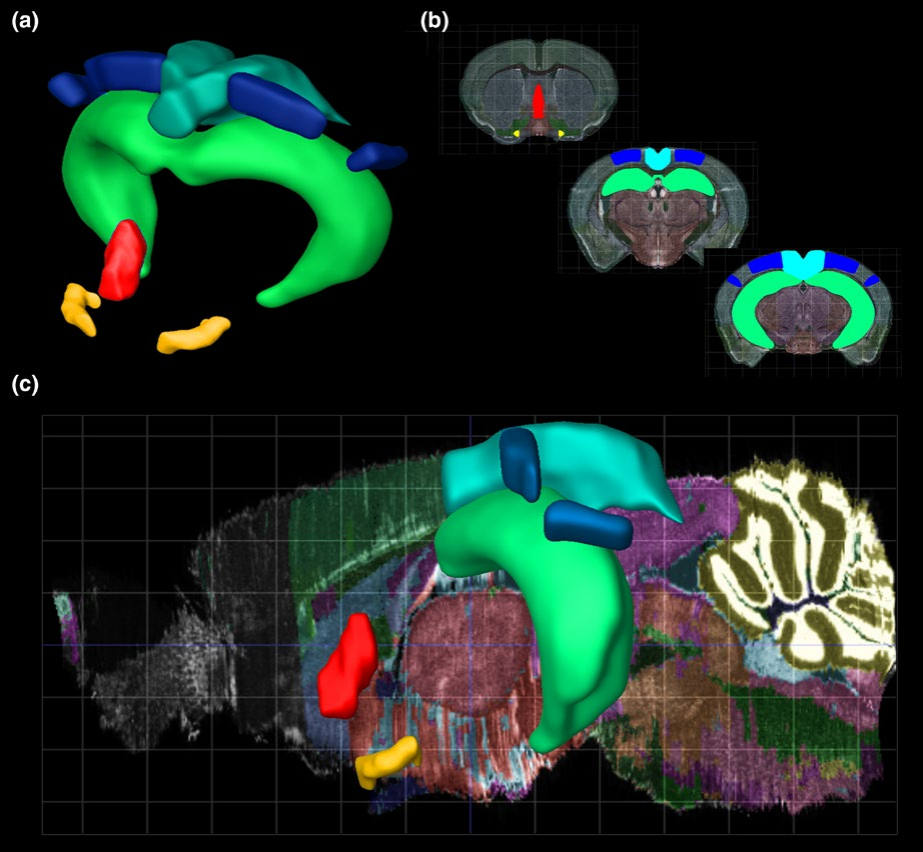
Anatomical location of the spatial navigation network. (a) 3D, (b) coronal and (c) sagittal views of the mouse brain highlighting the locations of medial septum (red), nucleus basalis magnocellularis (yellow), hippocampus (green), posterior parietal cortex (blue) and retrosplenial cortex (teal). Image credit: Allen Institute

Given the complexity of the process and the different ways by which navigation can be implemented, it is unsurprising that neuroimaging and lesion studies have identified an intricate network of structures involved, including the hippocampus, entorhinal cortex, parahippocampal gyrus, medial and right inferior parietal cortex, regions within prefrontal cortex, cerebellum, parts of the basal ganglia, posterior cingulate cortex and retrosplenial cortex (RSC; Guterstam, Björnsdotter, Gentile, & Ehrsson, 2015; Iaria, Chen, Guariglia, Ptito, & Petrides, 2007; Ito, Zhang, Witter, Moser, & Moser, 2015; Maguire et al., 1998; Rochefort, Lefort, & Rondi‐Reig, 2013). Hereafter, we will direct our focus on the differential role of the hippocampus, the posterior parietal cortex (PPC) and the RSC in spatial navigation, together which areas seem to form a network that may serve as the anatomical bases of flexible integration of allocentric and egocentric information. Next, we will review the structural properties of cholinergic basal forebrain (BF) afferents to these cortical structures originating from the medial septum (MS) and the nucleus basalis magnocellularis (NBM; Figure 1). Finally, we will summarize the functional evidence provided by lesion, microdialysis and pharmacology studies on the role of these BF to cortex pathways in controlling different aspects of spatial cognition. Although discussing other brain areas are beyond the scope of this review, this does not diminish their relevance for navigation (see for example (Mizumori, Puryear, & Martig, 2009; Chersi & Burgess, 2015) on the importance of striatal circuits in spatial navigation).

## 2 THE ROLE OF THE HIPPOCAMPUS, POSTERIOR PARIETAL AND RSC IN ALLOCENTRIC AND EGOCENTRIC REPRESENTATION OF SPACE

### 2.1 The hippocampus

The medial temporal lobe is a key structure in the spatial domain of cognition, for navigation as well as encoding and retrieval of spatial memory. This includes the anatomical areas of the hippocampal region (hippocampus proper, dentate gyrus and subicular complex) and the adjacent cortex (perirhinal, entorhinal, and parahippocampal cortices; Aggleton, 2012; Fernández‐Ruiz & Oliva, 2016; Lavenex & Amaral, 2000; Squire & Zola‐Morgan, 1991). The literature in this field is vast and multidisciplinary. Here we only provide an overview pertinent to our focus of cholinergic modulation of hippocampal function in the context of spatial learning and memory; for more extensive summaries and thought‐provoking reading please refer to (Eichenbaum et al., 2016; Lisman et al., 2017; Moser, Moser, & McNaughton, 2017). As stated by (O'Keefe & Nadel, 1978), “we shall argue that the hippocampus is the core of a neural memory system providing an objective spatial framework within which the items and events of an organism's experience are located and interrelated”. Hippocampus indeed seems to represent spatiotemporally coincident elements of a context, creating allocentric cognitive maps which are then used to guide exploration, plan navigation and interpret the current state of the world (Schiller et al., 2015).

Tasks requiring either spatial memory or navigation based on allocentric cues generally engage the hippocampus (Hartley, Maguire, Spiers, & Burgess, 2003; Iaria, Petrides, Dagher, Pike, & Bohbot, 2003; Kumaran & Maguire, 2005; Maguire et al., 1998) and are impaired in patients with hippocampal brain damage (Corkin, Amaral, González, Johnson, & Hyman, 1997; Feigenbaum & Morris, 2004; Guderian et al., 2015; Hartley et al., 2007; Holdstock et al., 2000; Scoville & Milner, 1957). Spatial navigation becomes gradually impaired during ageing. A recent study (Konishi, Mckenzie, Etchamendy, Roy, & Bohbot, 2017) found that the age‐related impairment of spatial memory was correlated with decreased hippocampal volume, while better general cognitive functions were associated with superior wayfinding abilities and increased use of hippocampus‐dependent spatial strategies.

A specific loss of cholinergic innervation of the hippocampal formation has been hypothesized as a leading cause for age‐related memory decline both in normal ageing and Alzheimer's patients (Arendt & Bigl, 1986; Gallagher & Colombo, 1995; Whitehouse, Price, et al., 1982). It was established that the extent of cognitive impairment was correlated with loss of BF cholinergic neurons from post‐mortem human brain tissue samples (Arendt & Bigl, 1986; Bowen, Smith, White, & Davison, 1976; Iraizoz, de Lacalle, & Gonzalo, 1991; Perry et al., 1978). Moreover, functional alterations of cholinergic activity might precede morphological degeneration (Palop & Mucke, 2016; Schliebs & Arendt, 2011); however, a direct support for specific cholinergic dysfunction before axonal degeneration and plaque formation is lacking. Given the well‐known age‐associated impairment of the medial septal cholinergic neurons (Schliebs & Arendt, 2011), this raises the possible causal involvement of diminishing cholinergic innervation in impaired hippocampal spatial memory during ageing.

Although the hippocampus is certainly fundamental for allocentric navigation, it has recently been proposed that such tasks might be implemented by a broader network (Ekstrom, Arnold, & Iaria, 2014). Indeed, analysing the trajectory of amnestic patients with medial temporal lobe damage in a virtual analogue of the Morris water maze (MWM), it was found that they retained the ability to acquire and utilise coarse spatial maps, with partial allocentric memory (Kolarik, Baer, Shahlaie, Yonelinas, & Ekstrom, 2018; Kolarik et al., 2016).

In rodents, lesions to the hippocampus impairs navigational tasks that require the processing of environmental cues or exploration of new locations, while simple stimulus‐response learning remains intact (Cohen, LaRòche, & Beharry, 1971; Morris, Garrud, Rawlins, & O'Keefe, 1982; Olton, Walker, Gage, & Johnson, 1977). However, the exact nature of the impairment and whether it is the same across rodents and humans remains less clear. Clark and colleagues used a version of the MWM that allowed the animal to proceed directly to the hidden platform using a beacon (Clark, Broadbent, & Squire, 2007). However, the rats first had to select the correct beacon from four identical objects based on distal cues. After the lesion, animals were unable to reach the platform, as expected if the hippocampus is necessary to process distal cues. However, contrary to the expectation that rats would look for the platform near the beacons, they did not show an indication of maintaining this concept, suggesting a broader memory impairment not restricted to spatial information. Additionally, while humans with hippocampal lesions retain spatial memories acquired remotely showing a temporally graded retrograde amnesia, lesioned animals are also impaired on recently acquired memories despite extensive training (Clark, Broadbent, & Squire, 2005). This could be resolved by raising and training the animals in an enriched environment, where they could acquire an allocentric spatial representation that survived hippocampal damage, confirming that extensive premorbid experience leads to spatial representations that are independent of the hippocampus (Winocur, Moscovitch, Fogel, Rosenbaum, & Sekeres, 2005). These spatial representations, however, became more schematic and could not support flexible navigation, e.g. choosing an alternative route in the presence of an unexpected obstacle (Winocur, Moscovitch, Rosenbaum, & Sekeres, 2010), similarly to human patients in a complex environment (Maguire, Nannery, & Spiers, 2006).

Crucial support for the navigational role of the hippocampal formation has come from electrophysiology experiments. Our current understanding of how the brain encodes spatial information has been shaped by the discovery of rodent place cells (O'Keefe & Dostrovsky, 1971), head direction cells (Taube, Muller, & Ranck, 1990), grid cells (Hafting, Fyhn, Molden, Moser, & Moser, 2005), conjunctive grid‐head direction cells (Sargolini et al., 2006), border cells (Solstad et al., 2008) and most recently speed cells (Kropff, Carmichael, Moser, & Moser, 2015), while other specific representations may be present (e.g. see Diehl, Hon, Leutgeb, & Leutgeb, 2017). In the last decade, analogous representations has been discovered in humans (Doeller, Barry, & Burgess, 2010; Ekstrom et al., 2003; Julian, Keinath, Frazzetta, & Epstein, 2018; Killian, Jutras, & Buffalo, 2012; Lee et al., 2017; Miller et al., 2013; Nadasdy et al., 2017; Nau, Navarro Schröder, Bellmund, & Doeller, 2018).

Place cells are hippocampal principal neurons that preferentially fire at given locations of the environment (‘place field’), thus encoding spatial information that collectively allow the reconstruction of the exact location of the animal (Chen, Andermann, Keck, Xu, & Ziv, 2013; O'Keefe & Dostrovsky, 1971; Olypher, Lánský, Muller, & Fenton, 2003; Skaggs, McNaughton, & Gothard, 1993). Moving across space, place cells are activated in a sequence representing a path that may be reactivated during sleep for memory consolidation (de Lavilléon, Lacroix, Rondi‐Reig, & Benchenane, 2015; O'Neill, Senior, Allen, Huxter, & Csicsvari, 2008; van de Ven, Trouche, McNamara, Allen, & Dupret, 2016) or during wakefulness to recall environmental features and plan actions (Ólafsdóttir, Carpenter, & Barry, 2017; Pfeiffer & Foster, 2013; van der Meer, Johnson, Schmitzer‐Torbert, & Redish, 2010; Wu, Haggerty, Kemere, & Ji, 2017). This organisation appears to be the default processing scheme of the hippocampus that extends beyond spatial navigation and creates sequential representations of non‐spatial features, probably serving memory‐guided behaviour in general (Allen, Salz, McKenzie, & Fortin, 2016; Aronov, Nevers, & Tank, 2017; Pastalkova, Itskov, Amarasingham, & Buzsaki, 2008). Distal visual and non‐vestibular self‐motion cues can provide enough spatial information to create place fields as observed in virtual reality (VR), but in natural environments where vestibular and other sensory cues are also present, a more robust hippocampal activity was observed (Ravassard et al., 2013). Thus place field representations likely rely on a combination of entorhinal inputs to hippocampus that include grid, head direction and border cells. Grid cells, like place cells, fire at specific locations of the environment, but they have multiple firing fields that form a triangular grid, and are located in clusters in medial entorhinal cortex and in pre‐ and para‐subiculum (Boccara et al., 2010; Fyhn, Hafting, Treves, Moser, & Moser, 2007; Hafting et al., 2005; Heys, Rangarajan, & Dombeck, 2014). Each head direction cell fires preferentially when the animal is facing a certain direction, thus head direction cells represent allocentric heading independent of location. They are found in the dorsal pre‐subiculum and entorhinal cortex, but also in other areas including the anterior dorsal thalamic nucleus and the RSC (Taube, 2007; Taube et al., 1990). While the head direction system seems to be essential for place cell stability (Calton et al., 2003; Harland et al., 2017), selective elimination of the grid input while retaining hippocampal place fields appears to be possible (Koenig, Linder, Leutgeb, & Leutgeb, 2011). However, how these diverse input patterns are integrated in hippocampal circuits to give rise to spatial and non‐spatial codes remains elusive (Danielson et al., 2016; Lovett‐Barron et al., 2012; Poucet et al., 2013).

Hippocampal theta oscillation (4–12 Hz) is generated by a subcortical network in which the MS likely plays a crucial pacemaker role (Fuhrmann et al., 2015; Hangya, Borhegyi, Szilagyi, Freund, & Varga, 2009; Mamad, McNamara, Reilly, & Tsanov, 2015). It has been linked to cognitive and other processes (Korotkova et al., 2018) among which exploration (Gangadharan et al., 2016), moving and running, with a direct correlation to speed, (Bender et al., 2015; Sheremet, Burke, & Maurer, 2016) and memory consolidation (Boyce, Glasgow, Williams, & Adamantidis, 2016) are important to spatial navigation. Indeed, septally induced theta rhythm is thought to carry linear velocity information, since this structure has speed cells, its activation/deactivation can initiate/stop locomotion (Fuhrmann et al., 2015), and paces theta in correlation with speed (Tsanov, 2017). Combining the cognitive map with movement information may allow updating the estimate of self‐position while moving; indeed, speed modulation of hippocampal theta frequency correlates with spatial memory performance in a spatial alternation task (Richard et al., 2013). This process may be mediated both by place and grid cells that could integrate spatial signals and velocity‐dependent theta waves both in rodents (Chen, King, Burgess, & O'Keefe, 2013) and humans (Bohbot, Copara, Gotman, & Ekstrom, 2017). Moreover, septal inactivation abolishes theta and disrupts grid cell coding (Brandon et al., 2011; Koenig et al., 2011), causes deficit in spatial working memory (Ma et al., 2009) and impairs rats’ ability to estimate linear distances based on self‐motion information (Jacob, Gordillo‐Salas, et al., 2017), while hippocampal place fields are maintained (Mizumori, McNaughton, Barnes, & Fox, 1989). Selective ablation of septal cholinergic neurons reduced the amplitude and spectral power of theta oscillation without eliminating it (Lee, Chrobak, Sik, Wiley, & Buzsáki, 1994; Rastogi, Unni, Sharma, Laxmi, & Kutty, 2014), while cholinergic stimulation enhanced hippocampal theta (Vandecasteele et al., 2014). Cholinergic M1 receptors located on hippocampal pyramidal neurons might be relevant in this process, being critical for hippocampal synaptic plasticity, theta generation and spatial memory performance in a Y‐maze (Gu, Alexander, Dudek, & Yakel, 2017). At the same time, the potential role of local intra‐septal connections has also been emphasized (Dannenberg et al., 2015; Yang et al., 2014; Zant et al., 2016).

### 2.2 The posterior parietal cortex

The PPC occupies the caudal part of the lateral cortex between the primary somatosensory area and the parieto‐occipital sulcus. It is generally regarded an associational cortical region, combining inputs from sensory cortices of multiple modalities with top‐down prefrontal inputs and buttom‐up subcortical proprioceptive and vestibular signals (Whitlock, 2017).

Posterior parietal cortex is involved in representing bodily position and spatial orientation, which are key features for understanding its role in navigation. In one of the first relevant studies, subjects familiarised with a complex virtual town had to navigate to an unseen target area while they underwent positron emission tomography (Maguire et al., 1998). Along with the hippocampus, parietal cortex was found to be activated when subjects had to perform a sequence of turns to reach a target, irrespective of whether they were performing difficult way‐finding tasks or simply following arrows towards the goal. Thus the parietal cortex may compute the correct body turns to enable navigating along a route, suggesting it has a crucial role in route creation using proximal‐egocentric cues, in contrast to the hippocampus that mainly represents allocentric maps.

Route learning using proximal salient cues was subsequently associated with a network of structures including the PPC, left medial frontal gyrus and left RSC. Subjects learnt to navigate in a VR maze with several landmarks at the crossroads; posterior inferior parietal regions showed increasing activation across sessions, correlated with behavioural measures of route expertise (Wolbers, Weiller, & Büchel, 2004). In an elegant study, London taxi drivers were imaged while navigating passengers in a detailed, topographically accurate videogame reproduction of the British capital. Parietal cortex was activated when the cab driver decided to change his route adapting it to environmental contingencies (e.g. change to a faster lane), confirming the role of PPC in ego‐centric route planning (Spiers & Maguire, 2006). Selective representation of navigationally salient egocentric information was recently found in the precuneus, part of the posterior medial parietal cortex (Chadwick, Jolly, Amos, Hassabis, & Spiers, 2015).

Similar conclusions were drawn from studying the consequences of PPC lesions. Patients with parietal lesions due to infarction had to navigate through a VR park (i.e. an open environment rich in landmarks) and a VR maze (i.e. a series of identical corridors and intersections) to reach a virtual gold pot; they were impaired on the latter, further corroborating that the impacted parietal area might be involved in processing egocentric cues (Weniger, Ruhleder, Wolf, Lange, & Irle, 2009). In another study, patients could recall a detailed image of their home city and make distance and proximity judgments but could not navigate between known locations or determine the correct sequence of landmark positions (Ciaramelli, Rosenbaum, Solcz, Levine, & Moscovitch, 2010). These results further suggest that the PPC is crucial for accessing remote spatial memories within an egocentric reference frame that enables both navigation and re‐experiencing.

Parallel to the above studies of parietal damage in human patients, rats with PPC lesions were found to be impaired on navigation based on proximal but not distal cues in the MWM (Save & Poucet, 2000). A partial explanation was found later by the same group, observing that rotation of proximal cues in an open field could not elicit a consequential shift in all hippocampal place fields after parietal lesions (Save, Paz‐Villagran, Alexinsky, & Poucet, 2005). These finding suggest a functional importance of the PPC in processing the local spatial frame of reference.

A finer‐grained insight on the complex role of this area may be gleaned from electrophysiological studies. PPC neurons were recorded in rats navigating in a maze that allowed reaching the goal through different routes (Nitz, 2006). Neuronal discharge reflected a combination of movement direction, spatial position and behavioural information such as left and right turns, often scaled by path segment size, suggesting a relationship with route progression. PPC neurons were also recorded while rats were traversing a squared spiral track (Nitz, 2012). Firing activity could simultaneously encode three frames of reference (segments, loops and routes), and could thus discriminate analogous segment positions in different loops while still representing reoccurring patterns across segments and loops; therefore, the PPC may help process reference frames for route computing. Another experiment confirmed that PPC is tuned both to allocentric and egocentric reference frames. Some neurons encoded the egocentric position of a light cue toward which the rat had to move, while others represented this conjunctively with the animal's head direction, consistent with the role of this area in orienting the body for goal‐directed route progression (Wilber, Clark, Forster, Tatsuno, & McNaughton, 2014). Single‐ and multi‐unit activity revealed that PPC is also tuned to self‐motion (Whitlock, Pfuhl, Dagslott, Moser, & Moser, 2012; Wilber, Skelin, Wu, & McNaughton, 2017). Activity patterns were found to reoccur in post‐experience sleep in a compacted fashion, temporally coordinated with hippocampal reactivation, suggesting a role in memory consolidation during sleep (Wilber et al., 2017). The role of PPC neurons in encoding position and heading has also been shown recently in head‐fixed mice performing a two‐alternative forced‐choice visual detection task by walking in a virtual T Maze (Krumin, Harris, & Carandini, 2017). The decisions of the animal were typically evident before the mouse reached the fork: the final choice could be predicted from the heading angle with increasing accuracy as the mouse reached the end of the main corridor. PPC neurons appeared to be selective for specific combinations of the animal's position and of its heading angle (“position‐heading field”).

### 2.3 The retrosplenial cortex

The RSC is a transition area in the posterior cingulate region that links limbic memory areas such as the hippocampus and cortical areas relevant to spatial navigation and behavioural processing of the dorsal stream coordinating visual and motor information (Miller, Vedder, Law, & Smith, 2014). RSC and PPC are densely interconnected, and are thought to cooperate in coordinating egocentric and allocentric information processing (Clark, Simmons, Berkowitz, & Wilber, 2018).

Patients with damages to the area, typically as a consequence of cerebral haemorrhages or tumour in the splenium of the corpus callosum, consistently show difficulty acquiring new information and retrieving recent autobiographical memories (Gainotti, Almonti, Di Betta, & Silveri, 1998; Osawa, Maeshima, Kubo, & Itakura, 2006; Valenstein et al., 1987). This has recently been confirmed in primates by within‐subject comparison of memory performance before and after controlled retrosplenial cortical lesions (Buckley & Mitchell, 2016). Damage involving the RSC can also cause a selective topographical disorientation: in most of these cases, patients recognize familiar landmarks or visual scenes but fail to describe routes between locations, draw a path or navigate efficiently even in familiar environments or learn to navigate in novel settings, indicating that they are unable to derive directional information from landmark cues (Greene, Donders, & Thoits, 2006; Ino et al., 2007; Maeshima et al., 2001; Maguire, 2001; Takahashi, Kawamura, Shiota, Kasahata, & Hirayama, 1997). Notably, patient TT, a former London taxi driver with bilateral hippocampal damage, was impaired at navigating in familiar and novel environments but, unlike patients with RSC lesions, could maintain a sense of direction and his ability to orient in familiar environments (Maguire et al., 2006). Studies using fMRI support the prominent role of RSC in processing the spatial relationships of landmarks, especially in connection with orientation in a novel environment (Auger, Mullally, & Maguire, 2012; Auger, Zeidman, & Maguire, 2015, 2017; Dilks, Julian, Kubilius, Spelke, & Kanwisher, 2011). Moreover, RSC is involved in processing heading direction (Marchette, Vass, Ryan, & Epstein, 2014; Shine, Valdés‐Herrera, Hegarty, & Wolbers, 2016) and spatial position on the basis of self‐motion cues or path integration (Sherrill et al., 2013, 2015; Wolbers & Büchel, 2005). Thus, it appears that the RSC supports allocentric representations by processing the stable features of the environment and their spatial relationships, but also enables us to localise ourselves in the environment. Along these lines, in a seminal review article Byrne et al. suggested that RSC is a “translational” area, transforming allocentric representations into egocentric representations and vice versa, allowing the formation of a comprehensive and complete spatial map (Byrne, Becker, & Burgess, 2007).

In rodents, lesions to the RSC impaired allocentric spatial memory in the MWM (Czajkowski et al., 2014; Sutherland, Whishaw, & Kolb, 1988; Vann & Aggleton, 2002), while overexpressing CREB in RSC resulted in spatial memory enhancements (Czajkowski et al., 2014). In addition, tests relying on path integration, integration of egocentric and allocentric information or switching between the two were also affected (Elduayen & Save, 2014; Nelson, Hindley, Pearce, Vann, & Aggleton, 2015; Nelson, Powell, Holmes, Vann, & Aggleton, 2015; Pothuizen, Davies, Aggleton, & Vann, 2010).

Combining a head‐fixed locomotion assay with Ca^2+^‐imaging in mouse RSC, a population of neurons located predominantly in superficial layers showed activity resembling that of hippocampal CA1 place cells during the same task, while they fired in sequences during movement showing firing fields that form a sparse, orthogonal code correlated with spatial context (Mao, Kandler, McNaughton, & Bonin, 2017). In another study, rats were trained on a T‐maze task in which the reward location was explicitly cued by a flashing light and RSC neurons were recorded as the rats learned. Most RSC neurons rapidly encoded the light cue, and this representation was not sensitive to the location of the light. However, some neurons encoded also the reward and its location, and they showed distinct firing patterns along the left and right trajectories to the goal (Vedder, Miller, Harrison, & Smith, 2016).

Head‐direction cells (HD), while present in other cerebral areas such as the anterior thalamus, striatum, entorhinal cortex and subiculum, represent about 8% of the RSC population, equally distributed across the granular and dysgranular layers. Some of these cells’ activity is modulated by the velocity of locomotion, while others are tuned to particular combinations of location, direction and movement (Chen, Lin, Green, Barnes, & McNaughton, 1994; Cho & Sharp, 2001). Those cells are reciprocally connected with the antero‐dorsal thalamus and influence HD firing to preferred direction (Clark, Bassett, Wang, & Taube, 2010). Interestingly, when rats were exploring two connected compartments containing landmarks in reversed orientation, causing conflict between global and local directional cues, some neurons in the dysgranular layer fired facing one direction in one chamber and the opposite in the other. This indicates that local environmental cues could prevail over head direction information in some neurons, eventually allowing association or dissociation of landmark cues from the head direction signal (Jacob, Casali, et al., 2017).

Recording from rats also confirmed the relevance of RSC in path integration and the integration of allocentric and egocentric elements. Neurons were recorded while the animal was traversing a route that required a specific sequence of body turns depending on the direction in which it was running in a room rich in distal environmental cues. The animals were making specific turn sequences (route‐based frames) while exposed to distal visual cues (allocentric frame). A population of neurons was found to code the animal's allocentric position in conjunction with the progress through the current route as well as left vs right turning behaviour (Alexander & Nitz, 2015). More recently, the same authors also found populations of RSC neurons that encoded route‐segments as well as the relative position of these segments within an allocentric framework (Alexander & Nitz, 2017). Rats were trained to navigate a track with a recursive structure; some neurons exhibited periodic activation patterns repeated across similarly shaped route segments, while a larger population exhibited periodicity over the full route, defining a framework for encoding sub‐route positions relative to the whole. This hints at the involvement of RSC in the extraction of path components and coding their spatial relationships.

## 3 BASAL FOREBRAIN INPUTS TO THE HIPPOCAMPUS AND CORTICAL AREAS OF THE NAVIGATION SYSTEM

### 3.1 Organization of the cholinergic BF projection system

Acetylcholine (ACh) is implicated in a wide range of cognitive processes such as arousal (Buzsàki & Gage, 1989; Kasanuki et al., 2018; Papouin, Dunphy, Tolman, Dineley, & Haydon, 2017), attention (Howe et al., 2017; Urban‐Ciecko, Jouhanneau, Myal, Poulet, & Barth, 2018), sensory processing (Eggermann, Kremer, Crochet, & Petersen, 2014; Froemke, Merzenich, & Schreiner, 2007; Pinto et al., 2013), reinforcement expectation (Hangya, Ranade, Lorenc, & Kepecs, 2015), reward and addiction (Shin, Adrover, & Alvarez, 2017; Siciliano, McIntosh, Jones, & Ferris, 2017). ACh is also involved in learning and plasticity: septal cholinergic inputs are capable of modulating different types synaptic plasticity in hippocampus with remarkable temporal precision, coordinating presynaptic and postsynaptic activities (Gu, Lamb, & Yakel, 2012; Gu & Yakel, 2011); cholinergic inputs to sensory cortices cause receptive field reorganizations (Froemke et al., 2007) and mediate novel encoding of non‐sensory information (Chubykin, Roach, Bear, & Shuler, 2013).

The vast majority of cholinergic input to neocortex, archicortex and subcortical structures arise from neurons located in the BF (Woolf, 1991). In primates these cells are intermingled with non‐cholinergic neurons and are distributed rostrocaudally in four partially overlapping groups, with different projection areas (Mesulam, Mufson, Levey, & Wainer, 1983; Mesulam, Mufson, Wainer, & Levey, 1983). This categorisation has been extended to rodents, although anatomical distinction of the specific nuclei appear more subtle (Coppola & Disney, 2018; Gorry, 1963). The medial septal nucleus (MS or CH1) and the vertical limb of the diagonal band of Broca (CH2 or VDB) send massive projections to the hippocampal formation, while the horizontal limb of the diagonal band of Broca (CH3 or HDB) projects to the olfactory bulb and frontal cortices. The fourth nucleus, CH4, also called NBM, consists of a heterogeneous ensemble of nucleus‐like structures, such as the sublenticular substantia innominata/extended amygdala (SI/EA), globus pallidus internus (GPi), internal capsule, nucleus ansa lenticularis, magnocellular preoptic nucleus (MCPO), nucleus basalis proper and according to most categorisations also the ventral pallidum (VP). These areas project to the neocortex in a medio‐lateral organisation: HDB/SI/VP neurons project to the medial frontal, cingulate, retrosplenial and visual cortex, while MCPO and nucleus basalis proper / GPi project to somatosensory, auditory and pyriform cortex. The lateral hypothalamus and basalateral amygdala are also densely innervated. This complicated nomenclature introduces some ambiguity in the literature; for instance, nucleus basalis sometimes refers to the entire posterior BF (CH3‐CH4), while in more refined treatments it designates the cholinergic system located along the internal capsule‐GPi and GP‐caudate borders (here termed nucleus basalis proper). Additionally, the boundaries between BF structures are not well established, hence projection targets of subregions vary across studies; nevertheless, there is strong consensus about the medio‐lateral projection topography along the anterio‐posterior axis of the BF.

The corticopetal projections from the BF cholinergic neurons (BFCN) have been extensively studied (Gielow & Zaborszky, 2017; Mesulam, Mufson, Levey, et al., 1983; Rye, Wainer, Mesulam, Mufson, & Saper, 1984; Zaborszky et al., 2015). Frontal cortex shows the highest cholinergic fibre density, followed by the occipital and parietal cortices. In contrast to cholinergic axons, GABAergic BF neurons appear to exclusively innervate inhibitory neurons (Zaborszky, Van Den Pol, & Gyengesi, 2012). The ratio of cholinergic to non‐cholinergic BF projection neurons systematically varies according to the cortical target area, lower in frontal (0.3 on average) than in posterior projecting areas (0.6). BFCN that target different cortical areas have a partial overlap in the rostro‐caudal extent of the BF, with a general topographical rule that neurons projecting to medial targets (e.g. frontal cortex) are located medially and rostrally, while those projecting to more lateral targets lay in more lateral and caudal parts (Zaborszky et al., 2015).

Elegant recent applications of monosynaptic retroviral rabies‐tracing started to provide a more complete picture on the organization of BF connectivity by cell type specific expression of the tracers using Cre driver lines (Do et al., 2016; Hu, Jin, He, Xu, & Hu, 2016). It was found that the striatum and hypothalamus provided the highest numbers of inputs to BFCNs (Hu et al., 2016), in line with previous anatomical studies (Cullinan & Záborszky, 1991; Henderson, 1997). The study also confirmed known projections from neuromodulatory centres such as ventral tegmental area and raphe nuclei, parts of amygdala, ascending brainstem input and innervation by olfactory areas (Gielow & Zaborszky, 2017). Cortical inputs to the BF in rats originate only in restricted regions of the cortex, including medial, lateral and orbitofrontal part of the prefrontal cortex, with a small contribution from the insular‐piriform cortices, arising mostly from deep cortical layers, with occasional labelling in layer 2/3. Interestingly, prefrontal fibres synapse exclusively on non‐cholinergic neurons, forming synaptic contact primarily with dendritic spines or small dendritic branches (89%); the remaining axon terminals established synapses with dendritic shafts (Zaborszky, Gaykema, Swanson, & Cullinan, 1997). Interestingly, Hu et al. found a small subset of inputs originating from frontal cortical areas directly onto cholinergic cells in mice. The distribution of the input is generally similar across the different BF cell types, while the output patterns are markedly different: for example, compared to the other cell types, the projection from BFCNs is stronger in the basolateral amygdala, hippocampus, and visual cortex but weaker in the lateral hypothalamus, lateral habenula, and the ventral tegmental area (Do et al., 2016). The recent development of a murine whole‐brain atlas of the cholinergic system using genetically labelled cholinergic neurons and whole‐brain reconstruction of optical images at 2‐μm resolution revealed cholinergic subgroups within the same area with different target region‐ and layer specificity (Li, Yu, et al., 2018).

Recently multiple cholinergic systems have been shown to co‐release other transmitters: cholinergic neurons of the striatum and medial habenula can release glutamate (Ren et al., 2011; Tritsch, Ding, & Sabatini, 2012); cholinergic BF neurons projecting to at least some neocortical areas co‐release GABA (Case et al., 2017; Saunders, Granger, & Sabatini, 2015; Saunders, Oldenburg et al., 2015), while those projecting to the basolateral amygdala appear to co‐release glutamate (Nickerson Poulin, Guerci, El Mestikawy, & Semba, 2006). Understanding the functional significance of releasing multiple transmitters by cholinergic and other neuromodulatory cell types requires further investigation; for recent reviews see (Ma, Hangya, Leonard, Wisden, & Gundlach, 2018; Tritsch, Granger, & Sabatini, 2016; Vaaga, Borisovska, & Westbrook, 2014).

Below we give a brief overview of the corticopetal projections of BF structures relevant for spatial learning and navigation, i.e. the MS and the NBM. These structures provide dense cholinergic innervation to the hippocampus, the RSC and the PPC, modulating their activity during spatial navigation.

### 3.2 The medial septum

Cholinergic cells constitute about 5% of the MS cell population, while the majority of neurons are GABAergic and glutamatergic (Gritti et al., 2006). Cholinergic cells are mainly found in the midline and the lateral parts of the MS, in decreasing numbers from anterior to posterior (Kiss, Borhegyi, Csaky, Szeiffert, & Leranth, 1997; Van der Zee & Luiten, 1994).

The major projection target of the MS is the hippocampal complex through the fimbra‐fornix pathway, mainly the hippocampus proper and the entorhinal cortex, with fewer efferents in perirhinal and postrhinal cortices (Gulyás, Acsády, & Freund, 1999; Kondo & Zaborszky, 2016). The MS also sends a limited number of axons to the medial habenula (Qin & Luo, 2009) and to retrosplenial, infralimbic and prelimbic cortices (Gaykema, Luiten, Nyakas, & Traber, 1990; Unal, Joshi, Viney, Kis, & Somogyi, 2015). The septo‐hippocampal pathway is the main source of ACh in the hippocampus (Dannenberg et al., 2015; Lewis & Shute, 1967; Nilsson & Björklund, 1992; Nilsson, Kalén, Rosengren, & Björklund, 1990; Vandecasteele et al., 2014), and provides widespread innervation of both principal cells and interneurons (Frotscher & Léránth, 1985). Contrary to the general view of mixed synaptic and non‐synaptic cholinergic signalling (Vizi & Kiss, 1998), it has recently been shown that the overwhelming majority of cholinergic terminals may establish synapses (Takács et al., 2018). More specifically, cholinergic fibres terminate in the stratum oriens of CA1 and CA3 (Matthews, Salvaterra, Crawford, Houser, & Vaughn, 1987), contacting pyramidal cells (Wainer et al., 1984), GABAergic interneurons (Leranth & Frotscher, 1989) and dentate granule cells (Nyakas, Luiten, Spencer, & Traber, 1987). Notably, cholinergic neurons were shown to activate astrocytes, which in turn excite GABAergic hilar interneurons, leading to inhibition of dentate granule cells (Pabst et al., 2016).

Parallel to the cholinergic projection, GABAergic BF neurons also send axons to CA1, CA3 and dentate gyrus, targeting different GABAergic interneuron types (Acsády, Halasy, & Freund, 1993; Freund, 1989; Freund & Antal, 1988; Gärtner, Härtig, Brauer, Brückner, & Arendt, 2001; Gulyás, Görcs, & Freund, 1990; Papp, Hajos, Acsády, & Freund, 1999; Takács, Freund, & Gulyás, 2008). The MS GABAergic subpopulation exhibiting the strongest theta rhythmicity, coined the Teevra cells, innervate CA3 interneurons selectively (Joshi, Salib, Viney, Dupret, & Somogyi, 2017). Septo‐hippocampal glutamatergic neurons (Colom, Castaneda, Reyna, Hernandez, & Garrido‐sanabria, 2005; Gritti et al., 2006) provide a hitherto less investigated projection to CA1 and CA3 where they target both pyramidal cells and interneurons, as well as interneurons of the dentate gyrus (Fuhrmann et al., 2015; Huh, Goutagny, & Williams, 2010; Manseau, Danik, & Williams, 2005; Sotty et al., 2003). Few neuropeptidergic efferents have also been reported to project from the lateral region of MS to CA2/3a (Peterson & Shurlow, 1992; Senut, Menetrey, & Lamour, 1989).

The RSC receives most of its cholinergic innervation from the NBM (Bigl, Woolf, & Butcher, 1982; Mesulam, Mufson, Levey, et al., 1983) and the DBB (Gonzalo‐Ruiz & Morte, 2000); however, a few cholinergic fibres originating in the MS were observed to innervate layer 1 and 3 of the RSC (Gage, Keim, Simon, & Low, 1994; Gonzalo‐Ruiz & Morte, 2000; Robertson, Baratta, Yu, & LaFerla, 2009; Tengelsen, Robertson, & Yu, 1992). While 75%–80% of MS neurons labelled retrogradely from RSC were choline‐acetyltransferase‐ (ChAT) positive, septal efferents also contained a GABAergic component (Freund & Gulyás, 1991) that mostly targeted various types of interneurons but in 7% pyramidal cells (Unal, Joshi et al., 2015).

### 3.3 The nucleus basalis magnocellularis

The NBM is most known for its large, multipolar cholinergic cells with extensive dendritic trees (Woolf, 1991), providing the main cholinergic input to the entire neocortical mantle (Price & Stern, 1983). They contribute 80%–90% of the NBM efferents in humans and primates (Raghanti et al., 2011). This proportion appears significantly lower (20%) in rodents, the rest provided by GABAergic and to a lesser degree glutamatergic neurons (Gritti, Mainville, Mancia, & Jones, 1997 but see Baskerville, Chang, & Herron, 1993), suggesting an upscaling of neocortical cholinergic projections in primates. The density of the cholinergic terminals varies across the six layers of cortex and depends on the cortical region studied; indeed, up to 13 possible patterns have been catalogued (Lysakowski, Wainer, Bruce, & Hersh, 1989). As a general pattern, there is usually a moderately high density of terminals in layers 1–3, low density in layer 4, again higher density in layer 5 and variable innervation of layer 6 (Eckenstein, Baughman, & Quinn, 1988). Cholinergic terminals form dendritic synapses, more frequently with shafts than spines and rarely with somata (Umbriaco, Watkins, Descarries, Cozzari, & Hartman, 1994), with a majority found on GABAergic neurons (Beaulieu & Somogyi, 1991). Cortical pyramidal neurons and PV+ interneurons are activated via muscarinic receptors upon weak cholinergic activation. Strong cholinergic inputs engage nicotinic receptors on layer 1 and non‐fast‐spiking layer 2/3 interneurons including those expressing vasoactive intestinal peptide (VIP+) (Alitto & Dan, 2012; Letzkus et al., 2011), probably leading to VIP‐mediated disinhibition (Lee, Kruglikov, Huang, Fishell, & Rudy, 2013; Pfeffer, 2014; Pi et al., 2013), but potentially also disynaptic inhibitory effects (Arroyo, Bennett, Aziz, Brown, & Hestrin, 2012).

Among the areas receiving cholinergic inputs from the NBM, the RSC (Bigl et al., 1982) and the PPC (Bucci, Conley, & Gallagher, 1999) are particularly relevant for the cognitive processes underlying spatial navigation. The RSC shows a distinctive cholinergic innervation pattern, with dense fibres in layer 1, sparse projections to layer 2/3, and strong innervation of layer 6. The PCC has a moderate and distributed cholinergic innervation, with some variation depending on the sub‐area considered (Lysakowski et al., 1989).

## 4 THE ROLE OF THE BF CHOLINERGIC SYSTEM IN SPATIAL NAVIGATION

### 4.1 Impairments of the cholinergic system and spatial navigation in Alzheimer's patients

Cognitive impairment in Alzheimer's patients has been linked to the net loss of cholinergic markers in the entorhinal cortex and BF neurons (Bartus, Dean, Beer, & Lippa, 1982; Geula & Mesulam, 1989; Whitehouse, Struble, Clark, & Price, 1982). In addition, BF atrophy was observed in Mild Cognitive Impairment (MCI) prodromal to AD (Grothe et al., 2010), suggesting that cholinergic dysfunction is central to the cognitive decline observed in pathological ageing. In normal ageing, cholinergic neurons show structural and functional abnormalities and a moderate cell loss (Schliebs & Arendt, 2011) that accompany minor cognitive deficits, suggesting a role for cholinergic decline in healthy ageing as well. In patients diagnosed with AD, spatial disorientation is one of the earliest symptoms present (McShane et al., 1998; Pai & Jacobs, 2004), already observed in MCI (Lithfous, Dufour, & Després, 2013) and gradually aggravating with disease progression (deIpolyi, Rankin, Mucke, Miller, & Gorno‐Tempini, 2007). Interestingly, even preclinical subjects, characterized by decreased Aβ_42_ levels in the cerebrospinal fluid prodromal to MCI, showed deficits in allocentric wayfinding but not egocentric route learning (Allison, Fagan, Morris, & Head, 2016). With disease progression, navigational difficulties seem to be connected with a diffuse brain damage in temporal and fronto‐parietal areas including RSC (Vlček & Laczó, 2014) thought to translate between egocentric and allocentric frames of reference (Alexander & Nitz, 2015; Sherrill et al., 2013). Specifically, navigation scores were correlated with grey matter density and glucose metabolism both in RSC and hippocampus (Pengas et al., 2010, 2012). In another study testing virtual as well as real navigation in a hospital lobby, AD but not MCI patients were impaired on a test of self‐orientation, requiring to indicate directions to scenes from the route (Cushman, Stein, & Duffy, 2008). Patients were also impaired on translating allocentric map representations to egocentric directions in a VR (Morganti, Stefanini, & Riva, 2013). In sum, anatomical and functional evidence points to an early involvement of the cholinergic system in learning deficits in Alzheimer's patients (Hampel et al., 2018). Nevertheless, human studies on cholinergic degeneration are still scarce. Specifically, more comprehensive anatomical data from post‐mortem samples establishing detailed inter‐relationships between cholinergic axonal degeneration, cell loss, plaque formation and clinical record of cognitive disabilities could reveal the course of pathology progression in more details.

### 4.2 The role of the cholinergic system in navigation: lesion studies

The role of BFCNs including both MS and nucleus basalis in spatial learning, navigation and spatial working memory was extensively studied in rodents by applying different techniques to introduce BF lesions. Non‐specific lesions were performed by injecting glutamatergic agonists ibotenic or quisqualic acid or AMPA, via administration of tetrodotoxin or by electrolysis (Kesner, Crutcher, & Measom, 1986; Page, Sirinathsinghji, & Everitt, 1995; Rashidy‐Pour, Motamedi, & Motahed‐Larijani, 1996; Steckler, Andrews, Marten, & Turner, 1993). Specific ablation of cholinergic neurons could be achieved by *in loco* injection of 192 IgG saporin (Lappi, Esch, Barbieri, Stirpe, & Soria, 1985; Wiley, Oeltmann, & Lappi, 1991), a ribosome‐inactivating protein covalently linked to an antibody against the p75 NGF receptor, specifically expressed by cholinergic neurons (but also Purkinje cells) in the adult brain (Book, Wiley, & Schweitzer, 1992; Dawbarn, Allen, & Semenenko, 1988). It should be noted however, that while small injections lead to incomplete lesions, large doses affected other cell types including parvalbumin‐expressing GABAergic neurons. Moreover, injections often spread into the striatum where they could theoretically impact cholinergic interneurons, whereas intraventricular injections destroyed cerebellar Purkinje cells and could affect norepinephrine levels in hippocampus (Heckers et al., 1994; Walsh et al., 1995). With histological verification rarely performed, these present complications in interpreting this body of literature (Hasselmo & Sarter, 2011; McGaughy, Everitt, Robbins, & Sarter, 2000; Wrenn & Wiley, 1998).

For assessing the impact of lesioning, the most widely applied test is the spatial version of the MWM, in which the animal has to learn and remember, using distal cues, the location of a hidden platform in a tank of opaque water. Since acquiring such memory takes multiple training sessions for the animal, the learning curve can be measured and quantified by changes in escape latency. To test the robustness of the memory, the platform is then removed and the time spent by the animal swimming near its previous location is measured during probe trial. Moderate spatial learning deficits were reported after selectively targeting the medial septal cholinergic neurons (Berger‐Sweeney et al., 1994; Frick, Kim, & Baxter, 2004; Hagan, Salamone, Simpson, Iversen, & Morris, 1988) while some of the studies failed to detect significant changes (Baxter, Bucci, Gorman, Wiley, & Gallagher, 1995; Baxter et al., 1996; Decker, Radek, Majchrzak, & Anderson, 1992; Dornan et al., 1997). More severe impairments on MWM were found when targeting the NBM cholinergic system, often in combination with the MS, or injecting the toxin in the ventricles (Miyamoto, Kato, Narumi, & Nagaoka, 1987; Nilsson et al., 1992; Berger‐Sweeney et al., 1994, 2001; Leanza, Nilsson, Wiley, & Björklund, 1995; Lehmann et al., 2000; Nieto‐Escámez, Sánchez‐Santed, & de Bruin, 2002 but see also Frick et al., 2004); nevertheless, the latter approach probably ablates parts of striatal cholinergic interneurons as well, thus affecting multiple parallel memory systems (discussed later in more details). Electrolytic (Miyamoto et al., 1987) and radiofrequency (Decker, Curzon, Brioni, & Arnerić, 1994) lesions of the whole MS caused a marked deficit in the spatial MWM, suggesting that non‐cholinergic neurons may also play an important role in spatial memory. This was further confirmed by selective lesions of GABAergic BF neurons (Lecourtier et al., 2011; Roland et al., 2014). As a potential synthesis, Wrenn and Wiley concluded that (i) learning and memory are only affected by ‘near‐complete’ lesions, probably because a relatively small proportion of cholinergic neurons can maintain basic functions; (ii) the mild impairments or lack of effects found in MS cholinergic lesions could partly be due to incomplete lesions and (iii) more complicated memory tasks show deficits more sensitively, while MWM can be considered fairly easy.

Another widely adopted apparatus to investigate spatial learning and working memory is the radial arm maze (RAM): here, using proximal or distal cues, the animal has to learn which arms are baited and must visit them only once. Cholinergic (Decker et al., 1992; Lehmann, Grottick, Cassel, & Higgins, 2003; Perry, Hodges, & Gray, 2001) or nonspecific (M'Harzi & Jarrard, 1992) lesions of the rat NBM, MS or both lead to a dramatic reduction of performance both in the spatial learning and cued version of this task. When exploring an environment featuring a choice of multiple arms (e.g. plus or T maze), rodents have the natural tendency to alternately enter each arm, avoiding consecutive visits of the same location. This behaviour relies on spatial working memory. Rodents show impairments in alternation behaviour after cholinergic ablation of the NBM or the MS, both separately and in combination (Chang & Gold, 2004; Dornan et al., 1997). In delayed‐matched‐to‐sample tasks, unlike spontaneous alternation, rodents learn to remember and re‐visit a previously sampled location. Also dependent on spatial working memory, animals with cholinergic lesions to the MS perform poorly on this task (Fitz, Gibbs, & Johnson, 2008; Johnson, Zambon, & Gibbs, 2002; Walsh, Herzog, Gandhi, Stackman, & Wiley, 1996). The MS cholinergic system is also involved in object location memory, evidenced by impaired performance on object location recognition in lesioned animals (Cai, Gibbs, & Johnson, 2012; Okada, Nishizawa, Kobayashi, Sakata, & Kobayashi, 2015).

Electrophysiological recordings support the idea of BFCNs being involved in spatial memory and navigation. First evidence was acquired from rats with fimbra‐fornix lesions, showing more dispersed, less reliable place fields in a familiar arena, which could be disrupted by alterations of the maze (e.g. 90° rotation; Shapiro et al., 1989). Place cell firing is preserved after MS lesions (Koenig et al., 2011; Mizumori et al., 1989) and resilient to mild disturbances by novel cues; however, repetitive exposure to new environments unmasked a decreased ability of the hippocampal place cell network to form new representations (Ikonen, McMahan, Gallagher, Eichenbaum, & Tanila, 2002). In accordance, place cell spatial representation were found to be less stable in a mouse model of AD (Mably, Gereke, Jones, & Colgin, 2017).

In summary, collective evidence suggests a fundamental role of the BFCNs in spatial cognition, supported by both lesion studies and clinical observations. Taking a closer look, however, one might discover a perplexing variability of outcomes of BF lesion studies in the ‘gold standard’ spatial MWM, with severe impairments emerging only in case of extensive, non‐specific lesioning. On the other hand, impaired performance was consistently reported in other tasks that likely represent higher cognitive load (RAM, T‐maze, novel object location). This suggests that BFCNs become necessary when the task demands spatial orientation along with more complex cognitive processing involving working memory and attention.

Perhaps the future of this line of studies lies in optogenetic and pharmacogenetic suppression of the cholinergic system, which could provide the necessary spatio‐temporal and cell type specificity. Such studies are still few but have so far demonstrated that BFCNs are necessary for the detection and discrimination of salient cues, probably serving cue‐guided responses (Gritton et al., 2016; Pinto et al., 2013). Notably, optogenetic inhibition of the cholinergic system also impaired the acquisition of learned fear behaviours (Jiang et al., 2016).

### 4.3 The role of cholinergic afferents inferred from pharmacology experiments

Acetylcholine acts both on G protein‐coupled metabtropic ‘muscarinic’ receptors (mAChRs, M1‐M5) and ionotropic ‘nicotinic’ receptors (nAChRs, pentamers of different types of α and β subunits). The most commonly expressed muscarinic receptors in the brain are M1 (activating), M2 and M4 (inhibiting) (Levey, Kitt, Simonds, Price, & Brann, 1991). Notably, presynaptic M2 receptors act as inhibitory auto‐receptors on cholinergic terminals and have been used as markers for cholinergic neurons (Brown, 2010). Most nAChRs in the mammalian brain are either α4β2 or α7, that lead to fast depolarisation through increased cation permeability, either enhancing transmitter release presynaptically or leading to excitatory postsynaptic responses, depending on localisation (Alkondon & Albuquerque, 2004; Dani & Bertrand, 2007). α7 receptors show lower affinity for nicotine, rapid desensitisation and fast kinetics compared to α4β2 (Giniatullin, Nistri, & Yakel, 2005). Two additional centrally expressed nicotinic receptors worth mentioning. First, the recently described α7β2 receptor has unique pharmacological and functional characteristic, being highly sensitive to functional inhibition by pathologically‐relevant concentrations of oligomeric, but not monomeric or fibrillar forms of amyloid β_1‐42_ (Aβ_1‐42_) (Liu et al., 2009; Wu et al., 2016). During the progression of AD, cholinergic inputs degenerate and the number of nAChRs in temporal areas decreases (Nordberg, 1994). Second, while there is generally little support for nicotinic postsynaptic effects on pyramidal neurons, as a notable exception, layer 6 pyramidal neurons express the relatively rare α5‐subunit together with β2 in heteromeric nicotinic receptors leading to nicotinic postsynaptic potentials and regulating short‐term plasticity. (Hay, Lambolez, & Tricoire, 2016; Hedrick & Waters, 2015; Verhoog et al., 2016) For detailed description on the distribution and differential impact of cholinergic receptors in the hippocampus and neocortex, see (Dannenberg, Young, & Hasselmo, 2017; Obermayer, Verhoog, Luchicchi, & Mansvelder, 2017).

Most pharmacological manipulations of the cholinergic system has been performed by systemic injection of the pharmacons, while fewer studies employed intracortical or intraventricular administration through implanted cannulae. In the following, we summarize the results and conclusions gained by applying cholinergic agonists and antagonists in behaving animals; for a more extensive review, see (Deiana, Platt, & Riedel, 2011; Everitt & Robbins, 1997; Haam & Yakel, 2017; McGaughy et al., 2000; Pepeu & Giovannini, 2010).

The muscarinic antagonist scopolamine has been widely used to induce “cholinergic amnesia” (Klinkenberg & Blokland, 2010) and has been shown to impair spatial navigation (Svoboda, Popelikova, & Stuchlik, 2017). Systemic injection of scopolamine either before the training or the test disrupted spatial learning and memory in the MWM (Huang et al., 2011); working‐ and short‐term memory performance was also impaired in RAM (Kay, Harper, & Hunt, 2010; Pilcher, Sessions, & McBride, 1997) and T‐maze experiments (Spowart‐Manning & van der Staay, 2004). When introducing the drug to well‐trained animals, there were usually no effects observed, suggesting that cholinergic demand varies during different stages of memory processing. While generally viewed as a muscarinic antagonist acting mostly via blocking M1 receptors (Burke, 1986), its specificity is questionable; indeed, it has been shown to increase ACh release through blocking presynaptic M2 auto‐receptors, leading to an upregulation of cholinergic effects under some conditions. For this property, scopolamine was widely used to enhance cholinergic efflux in ACh bioassay experiments (Phillis, 2005). In addition, it may partially affect nicotinic, glutamatergic and serotonergic receptors as well, further broadening the range of possible interpretations (Falsafi, Deli, Höger, Pollak, & Lubec, 2012; Lochner & Thompson, 2016). Nevertheless, the selective M1 antagonist pirenzepine exerted effects similar to those of scopolamine in spontaneous alternations in a Y maze, and this effect could be fully reversed by cN‐A‐343, a selective M1 agonist, but only partially by the nonselective muscarinic agonist oxotremorine (Ukai, Shinkai, & Kameyama, 1995). Consistently, the M1 agonist AF102B significantly reduced the age‐related cognitive deficit in rats, allowing them to learn platform location in the MWM at a time course comparable with young controls (Brandeis, Dachir, Sapir, Levy, & Fisher, 1990).

Nicotinic blockade also leads to impairments of spatial memory. Mecamylamine, a widely used non‐selective nicotinic antagonist disrupts learning and memory in the MWM; this impairment is additive but not synergistic with similar effects of scopolamine, suggesting at least partially independent mechanisms (Cozzolino et al., 1994; Decker & Majchrzak, 1992; Riekkinen, Sirviö, Aaltonen, & Riekkinen, 1990). Mecamylamine also reduces memory performance in the delayed‐match‐to‐sample version of the RAM (Maviel & Durkin, 2003). With a marked contrast to scopolamine, mecamylamine exerted its detrimental effects not only before training or testing but also by introducing post hoc (Brucato, Levin, Rose, & Swartzwelder, 1994; Levin, Castonguay, & Ellison, 1987). Similar results were obtained when applying more specific drugs such as dihydro‐β‐erythroidine (DH‐β‐E), a selective α4β2 antagonist or methyllycaconitine (MLA), specific for the α7 subunit (Andriambeloson, Huyard, Poiraud, & Wagner, 2014; Curzon, Brioni, & Decker, 1996) .

Consistent with the impairments induced by nicotinic antagonists, nicotine itself was found to have a beneficial effect on memory performance and cognition in general (Levin, McClernon, & Rezvani, 2006). Nicotine can partially rescue age‐related decline of spatial memory performance in MWM and RAM in mice (i) aged naturally (Levin & Torry, 1996; Socci, Sanberg, & Arendash, 1995), (ii) treated with AF64A, a neurotoxic derivative of choline that causes AD‐like cognitive impairments (Yamada, Furukawa, Iwasaki, & Ichitani, 2010), and (iii) after radiofrequency lesions of the MS (Decker et al., 1992). However, these beneficial effects depend on the dose and mode of application (Levin & Torry, 1996; Taylor, Bassi, & Weiss, 2005); for example, while acute injection of nicotine could improve the retention of the original position of an object in the novel object location (NOL) task (Melichercik, Elliott, Bianchi, Ernst, & Winters, 2012), chronic administration had an opposite effect (Kenney, Adoff, Wilkinson, & Gould, 2011). Chronic nicotine infusion, on the other hand, was found to improve memory performance in the RAM, suggesting that results may also differ depending on the exact behavioural paradigm of choice (Levin, Christopher, Weaver, Moore, & Brucato, 1999). Selective α7 or β4 ligands show similar effects. The selective α7 agonist DLPhtCho was able to reduce the scopolamine‐induced negative effect on MWM learning (Yaguchi, Nagata, & Nishizaki, 2009). Similarly, the selective β4 agonist SIB‐1553A, although ineffective in normal subjects, could reverse age‐related or scopolamine‐induced working memory deficit in a T maze task (Bontempi, Whelan, Risbrough, Lloyd, & Menzaghi, 2003; Bontempi et al., 2001).

There are considerably fewer studies that have investigated the effect of local, intracerebral drug applications. Scopolamine infusion in the dorsal hippocampus impaired acquisition and retention of spatial memory in the MWM (Herrera‐Morales, Mar, Serrano, & Bermúdez‐Rattoni, 2007). Interestingly, the M1 antagonist pirenzepine disrupted long‐term spatial memory but not acquisition, while the M2 antagonist BIBN‐161 had no effect; however, when applied in combination, they caused a complete impairment similar to the effect of scopolamine. Thus in contrast with systemic pirenzepine application mentioned above (Ukai et al., 1995), local infusion had a more defined effect, suggesting that recall but not learning is mediated by M1 receptors in dorsal hippocampus (Hagan, Jansen, & Broekkamp, 1987; Hunter & Roberts, 1988). Scopolamine injection to the ventral hippocampus decreased performance in the RAM (Kim & Levin, 1996). Similar impairments were observed when nicotinic antagonists (mecamylamine, MLA or DH‐β‐E) were injected to either ventral (Felix & Levin, 1997) or dorsal hippocampus (Nott & Levin, 2006). These manipulation were shown to affect the hippocampal place code: while recording in CA1, scopolamine or specific muscarinic antagonists pirenzepine or methoctramine (M2/M4‐antagonist) were infused locally through a dialysis probe. Scopolamine significantly reduced in‐field firing rates and increased the ratio of out‐of‐field to in‐field rates of place cells, and reduced the smoothness of the rate maps (Brazhnik, Borgnis, Muller, & Fox, 2004), consistent with systemic effects of scopolamine on spatial coding (Newman, Climer, & Hasselmo, 2014). The specific antagonists had limited effects in isolation, but reproduced these effects of scopolamine when applied in combination.

Intraseptal infusion of scopolamine impaired spatial learning in the MWM (Elvander et al., 2004) and memory performance in a T maze alternation task, in a dose‐dependent manner (Givens & Olton, 1990). Interestingly, the cholinergic agonist carbachol could also cause spatial memory impairments in MWM and delayed‐non‐match‐to‐sample tasks (Bunce, Sabolek, & Chrobak, 2004a, 2004b; Elvander et al., 2004), probably by facilitating encoding at the expense of retrieval, hence disturbing their balance. Indeed, carbachol prevented the retrieval of spatial representations of familiar environments when injected to CA1 (Sava & Markus, 2008). At the same time, encoding of new environments was facilitated specifically in aged rats, potentially due to the restoration of age‐impaired encoding processes. In line with these, the muscarinic agonist oxotremorine also improved spatial memory performance but only in old animals (Frick, Gorman, & Markowska, 1996; Markowska, Olton, & Givens, 1995).

Local application of scopolamine, mecamylamine or carbachol to NBM prevented spatial learning in MWM (Wilson, Munn, Ross, Harding, & Wright, 2009). However, it is hard to decipher whether these impairments were specific to spatial coding or at least partially a consequence of impaired attentional (Chudasama et al., 2004; Ljubojevic et al., 2018) or working memory processes (Croxson, Kyriazis, & Baxter, 2011; Ragozzino & Kesner, 1998; Sun et al., 2017; Yang et al., 2013).

In summary, conclusions based on local application of cholinergic drugs are roughly consistent with the results of systemic treatment, showing impairments of spatial memory encoding and retrieval by both nicotinic and muscarinic antagonists. Conversely, drugs that activate nicotinic receptors enhance the encoding of new information. These results should be considered with two caveats. Firsts, when drugs are applied systemically, it is impossible to exclude off‐target effects and the involvement of other structures. Indeed, these results are consistent with the nootropic effect of nicotine (Newhouse et al., 2012; Sarter, 2015) and amnestic effect of scopolamine (Klinkenberg & Blokland, 2010) that have been observed in other cognitive domains such as fear memory (Wilson & Fadel, 2017) or operant conditioning (Leach, Cordero, & Gould, 2013; Shi et al., 2013). Nevertheless, local delivery of muscarinic antagonists in dorsal or ventral hippocampus still had a negative impact on spatial memory. Second, BFCNs may co‐release ACh and GABA under certain circumstances, rendering BFCN lesions and anti‐cholinergic drug effects only partially comparable. Nonetheless, electrophysiological recordings of place cell and grid cell activity support the idea that the observed deficits are at least in part due to altered spatial processing and abnormalities of cholinergic activity.

### 4.4 Acetylcholine levels during navigation: microdialysis studies

Further insight about the role of the cholinergic system in spatial learning and memory was provided by microdialysis studies, in which ACh efflux is measured at a time resolution of seconds to minutes, depending on the technical details (König, Thinnes, & Klein, 2017). ACh levels were monitored in parietal cortex and hippocampus while rats were navigating through an open field for the first and second time (novel and familiar environment, respectively; Giovannini et al., 2001). During the first exploration a significant increase in cortical and hippocampal ACh release was observed, and when the animals were placed back in their home cage it slowly returned to basal levels. The exploration of the same arena in a second occasion caused a smaller and shorter lasting increase in ACh, suggesting a cholinergic component associated with novelty. When rats were trained in a RAM or a T maze alternation task, hippocampal ACh level was found to progressively increased during learning across days and the magnitude of change was positively correlated with spatial memory performance (Fadda, Cocco, & Stancampiano, 2000; Giovannini et al., 2001). Interestingly, this activation in trained animals not only persisted during performing the task, but was already present when rats were placed in a “waiting cage”. Naïve animals showed a smaller increase in both environments, indicating that increased neurotransmitter release was both due to the novel environment and task anticipation (Fadda, Melis, & Stancampiano, 1996). In rats performing the same task, parallel to the rise of hippocampal ACh levels, an even bigger increase of ACh efflux was observed in the 29ab sub‐region of the RSC, which persisted after the end of the task (Anzalone, Roland, Vogt, & Savage, 2009). Septal GABAergic lesion by GAT1‐saporin impaired hippocampal ACh efflux as well as memory performance in a delayed non‐match‐to‐position task (Roland et al., 2014).

Interesting insights were also gained from studies on a rodent models of diencephalic amnesia (i.e. the Wernicke‐Korsakoff Syndrome), the pyrithiamine‐induced thiamine deficiency (PTD). Rats in the PTD group were kept on a thiamine‐free diet and were given daily injections of pyrithiamine hydrobromide, eventually leading to seizures at which point thiamine was reintroduced. PTD rats showed lower hippocampal ACh increase and impaired performance both on a spontaneous alternation and a non‐match‐to‐position task. This was paralleled by a selective loss of MS/DB cholinergic neurons revealed by stereology (Roland & Savage, 2007; Savage, Roland, & Klintsova, 2007).

Parallel memory systems can compete or collaborate depending on the task both in animals and humans (Poldrack & Packard, 2003; White & McDonald, 2002). For instance, simple navigation problems like alternation in a T maze can be solved both applying an allocentric (place) and an egocentric (response) strategy. These are thought to be dependent on the hippocampus and the dorsal striatum, respectively. In a study from McIntyre and colleagues, both hippocampal and striatal ACh levels were monitored while rats were trained in a maze where the baited arm could be reached employing either of the strategies. Some rats used a spatial strategy while others a turning strategy; interestingly, this choice could be predicted by the ratio of hippocampal to striatal ACh efflux not only during the training, but even in the preceding baseline period (McIntyre et al., 2003). Another study from the same group showed that while hippocampal ACh levels were high from the beginning of the training and maintained at the same levels, striatal ACh efflux increased gradually, suggesting that there might be a gradual shift from allocentric to egocentric strategies during navigation (Chang & Gold, 2003). When rats were trained on a response strategy explicitly, they showed higher striatal ACh levels compared to learning a spatial version of the same maze (Pych, Chang, Colon‐Rivera, Haag, & Gold, 2005). In contrast, hippocampal ACh levels were more related to the abundance of sensory cues, suggesting that hippocampus may enhance learning a response strategy when extra‐maze cues are available.

This dissociation between striatal and hippocampal systems in spatial learning has also been demonstrated using the MWM. Animals that had received pre‐training lesions or expressed a dominant‐negative mutant of CREB in the dorsal striatum were impaired in learning the cued navigation task, but showed a faster learning on the spatial version, while animals lesioned in the dorsal hippocampus showed opposite effects, suggesting a competition between the striatal and hippocampal memory systems (Lee, Duman, & Pittenger, 2008). Consistent with these findings, PTD‐treated rats relied more on egocentric strategies and showed higher striatal ACh efflux compared to the controls (Vetreno, Anzalone, & Savage, 2008). However, a co‐operative rather than competitive interaction between these systems has been demonstrated in human patients of Huntington disease (Voermans et al., 2004). Evidence for synergistic effects were also demonstrated when lesions were performed after rats were trained both on spatial and cued navigation (Ferbinteanu, 2016). In this case, performance on both tasks was impaired after lesioning either the hippocampus or the dorsal striatum.

Therefore, while ACh release in hippocampus seems to be essential for spatial learning with allocentric cues, high cholinergic levels in tasks where no spatial strategy is obvious suggest that hippocampus is also involved in broader aspects of learning or performing the task in general, e.g. memory encoding, sustained attention and detecting changes in task contingencies that would require a strategy shift. Clarifying the relationship between cholinergic firing activity, ACh‐mediated postsynaptic currents and detectable extracellular choline levels could aid the interpretation of these data in the future. In addition, time course of receptor availability for synaptic and extrasynaptic ACh could help establish a link between pharmacology and microdialysis / voltammetry studies.

### 4.5 The role of cholinergic signalling in cortical processing

Electrophysiology studies have also shed light on how cholinergic innervation may influence cortical processing. Septo‐hippocampal afferents has been shown to control the amplitude of hippocampal theta oscillation relevant for spatial navigation and learning, without strongly affecting its frequency (Hasselmo, Hay, Ilyn, & Gorchetchnikov, 2002; Lee et al., 1994), the latter probably controlled by septal GABAergic pacemaker neurons (Borhegyi, Varga, Szilágyi, Fabo, & Freund, 2004; Hangya et al., 2009; Ujfalussy & Kiss, 2006). Theta oscillation was proposed to separate encoding and retrieval processes in the CA1, mediated by entorhinal and CA3 inputs, respectively (Hasselmo, Bodelón, & Wyble, 2002; Koene, Gorchetchnikov, Cannon, & Hasselmo, 2003). This model was later confirmed experimentally in an elegant closed‐loop stimulation study by Siegle and Wilson (Siegle & Wilson, 2014). In this process, cholinergic afferents are thought to enhance the encoding phase (Barry, Heys, & Hasselmo, 2012; Hasselmo, 2006; Hasselmo, Hay et al., 2002); indeed, cholinergic antagonists reduce theta‐gamma cross‐frequency coupling and the modulation of theta oscillation by animal speed (Newman et al., 2017). As an extension of the above model, theta oscillation was proposed to coordinate the integration of different sensory modalities for spatial navigation (Tsanov, 2017). Cholinergic neurons may exert their effects both directly on hippocampal interneurons and pyramidal cells and indirectly via the innervation of septo‐hippocampal GABAergic neurons (Dannenberg et al., 2015; Yang et al., 2014). Cholinergic manipulations influence the firing of both place cells and grid cells, possibly through presynaptic inhibition of excitatory and inhibitory synaptic transmission in the hippocampus and entorhinal cortex and the modulation of intrinsic currents in excitatory and inhibitory neurons (Dannenberg, Hinman, & Hasselmo, 2016).

Cholinergic afferents to the hippocampal CA1 were shown to be important for spike‐timing‐dependent plasticity (Seol et al., 2007). Relative timing of Shaffer collateral stimulation with respect to optogenetic activation of cholinergic fibres determined the form of plasticity in a time‐sensitive manner on the millisecond scale (Gu & Yakel, 2011). This effect required both pre‐ and postsynaptic processes (Gu et al., 2012) and involved α7 nicotinic as well as muscarinic receptors (Gu & Yakel, 2011; Yakel, 2012).

Little is known about how coordinated firing of BF cell assemblies may influence cortical processing and cognition. A series of papers from Nitz and colleagues demonstrated the formation of such assemblies in the nucleus basalis. These are orchestrated by local beta oscillations (Quinn, Nitz, & Chiba, 2010; Tingley et al., 2014) that appear to be implicated in learning processes (Quinn et al., 2010) and recruited in specific task phases during cognitive processing (Tingley et al., 2014). A correlated, temporally coordinated activation of posterior parietal cortical neuron populations was observed (Tingley et al., 2014), which may serve as a basis for BF modulation of PPC activity during spatial learning and navigation. In rough agreement, beta coherence between prefrontal cortex and PPC was found to be influenced by nucleus basalis cholinergic lesions in another study (Ljubojevic et al., 2018). In addition, nucleus basalis may exert a more global influence on the so‐called default mode network involving both the retrosplenial and the anterior cingulate cortex (Nair et al., 2018; Turchi et al., 2018). Nevertheless, how cholinergic control of cortical firing serves spatial learning and navigation will require further investigation.

## 5 CONCLUSIONS

**Figure 2:**
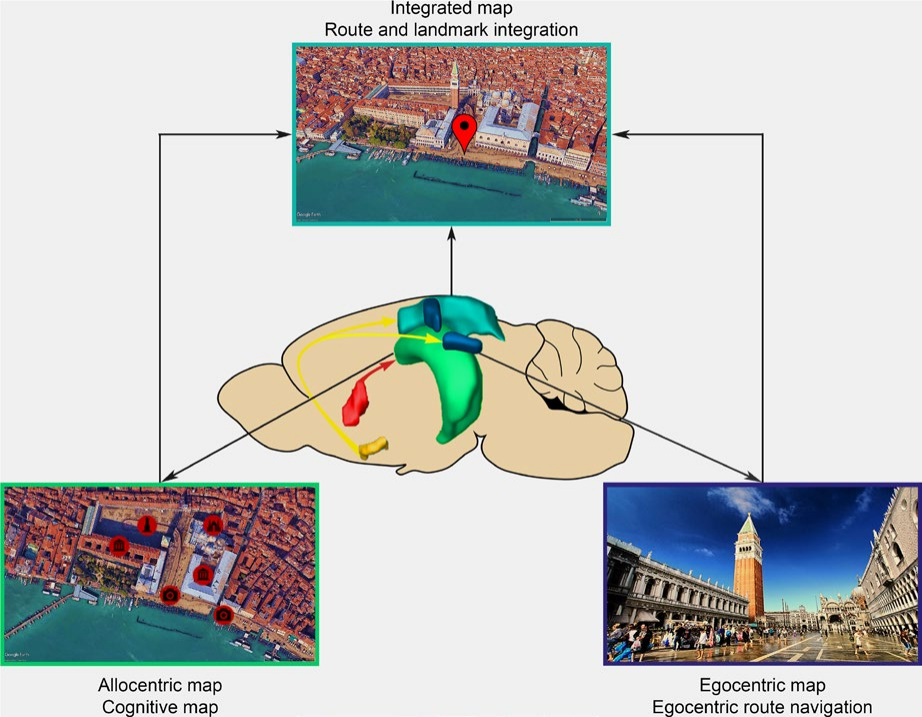
Key anatomical and functional relationships of the spatial navigation network. The basal forebrain nuclei send cholinergic efferents to the hippocampus and the cortex, regulating their activity. Hippocampus and posterior parietal cortex represent allocentric and egocentric information, respectively, which are then integrated in the retrosplenial cortex

The hippocampus, retrosplenial and posterior parietal cortices are fundamental to spatial navigation, necessary for the formation, integration and flexible use of egocentric and allocentric frames of reference to create routes towards distant goals. Hippocampus integrates environmental features into an allocentric cognitive map with associated meta‐information to guide exploration; PPC is crucial for egocentric route planning, while RSC integrates self‐based and world‐based reference systems to allow optimal navigation strategies (Oess, Krichmar, & Röhrbein, 2017; Figure 2).

Basal forebrain cholinergic neurons project to all of these structures. While lesion studies lead to variable results and a general uncertainty about the degree to which these innervations are necessary for navigation, pharmacology studies interfering with cholinergic receptors consistently reported impaired spatial memory encoding and retrieval, spatial working memory and navigation in general. Measuring hippocampal ACh efflux by microdialysis suggested that hippocampal ACh levels are strongly correlated with the employment of allocentric orientation and navigation strategies. Investigating the hippocampal place code after lesions of the cholinergic system showed maintained hippocampal place cells but impaired grid cell firing and decreased flexibility and resilience to environmental stress of spatial coding, suggesting a non‐trivial role of subcortical cholinergic inputs.

Thus, while we can claim that ACh has an important role in spatial cognition, we have little mechanistic understanding of the nature of this role, despite the tremendous effort put in cell type specific lesioning and pharmacology experiments. This might be attributed to methodological issues, which are related to four types of specificity: (i) spatial specificity: where do we intervene, (ii) temporal specificity: on what timescale does our manipulation exert its effect, (iii) cell type specificity: which neurons do we manipulate and finally (iv) behavioural specificity: how do we test spatial cognition.

Spatial specificity relies on anatomical knowledge. While the hippocampus is arguably the best characterized brain area as far as structure is concerned, much less is known about the relatively complex anatomy of the BF. For instance it is generally considered that the BF sends ‘parallel’ cholinergic, GABAergic and glutamatergic projections to cortical areas. However, recent studies suggest that the extent of this targeting may vary across BF subregions (Gielow & Zaborszky, 2017; Zaborszky et al., 2015) and it is generally not known whether different BF cell types of a given area have the same projection targets. In addition, there is little information on how input and output patterns change within the BF in a cell type specific manner as the few studies addressing this question has largely ignored the anatomical complexity of the BF so far (Do et al., 2016; Hu et al., 2016; Li, Yu et al., 2018). Moreover, local connections within the BF are only partially mapped (Do et al., 2016; Gielow & Zaborszky, 2017; Yang, Thankachan, McCarley, & Brown, 2017; Yang et al., 2014). Therefore, we expect that improving our anatomical understanding of BF structures, cell type and subregion specific inputs and outputs and differences in local connectivity using novel viral labelling techniques including monosynaptic rabies tracing or cTRIO (Beier et al., 2015; Schwarz et al., 2015) will improve our abilities of spatially specific targeting of the BF. New insights may also arise using promising alternatives to the 192‐IgG saporin lesioning such as the use of immunotoxin in transgenic animals (Okada et al., 2015). Optogenetic methods could further increase spatial specificity compared to the limited possibilities of local drug injections or localised lesioning approaches (Siegle & Wilson, 2014).

Multiple cognitive, homeostatic and other physiological processes operate in parallel at a large variety of temporal scales. For instance, spatial learning processes may be influenced by attention, arousal, task engagement, motivation, fatigue, and ultimately, wakefulness. These change at different times and with different speed, providing a chance to separate them conceptually. For this, temporal control over the experimental manipulations are needed (Solari, Sviatkó, Laszlovszky, Hegedüs, & Hangya, 2018), only partially provided by pharmacology and microdialysis and entirely lacking in lesion studies. Here we expect that the temporal precision of combined electrophysiology, optogenetics and high throughput rodent cognition assays will lead to significant breakthroughs in understanding how cholinergic activity can support multiple cognitive processes at different time scales (Buzsaki et al., 1988; Hangya et al., 2015; Sarter, Lustig, Howe, Gritton, & Berry, 2014; Tingley et al., 2014).

Cholinergic, GABAergic and glutamatergic neurons are intermingled in the BF (Gritti et al., 2006; Zaborszky, Pang, Somogyi, Nadasdy, & Kallo, 1999). Subtypes of these send projections to cortex and other areas, some co‐express other markers like somatostatin or calretinin (Gritti, Manns, Mainville, & Jones, 2003; Zaborszky et al., 2012), whereas a subset (or all?) cholinergic neurons co‐release GABA (Case et al., 2017; Saunders, Granger et al., 2015; Takács et al., 2018). Different cell types are often associated with different functions and tremendous insights has been gained by recording cell type specific activities or conducting cell type specific manipulations in the past. While classical methods as pharmacology or IgG‐saporin lesions were limited in their cell type specificity, modern optogenetic and chemogenetic tools opened a new avenue for cell type specific interrogation (Barry, Akopian, Cepeda, & Levine, 2018; Herman et al., 2016; Li, Zeng et al., 2018; Orr et al., 2015), likely delivering precious new information about cholinergic function in the near future.

Indeed these techniques have already helped demonstrate the role of ACh in modulating cortical activity and enhancing sensory detection and discrimination (Eggermann et al., 2014; Pinto et al., 2013; Rothermel, Carey, Puche, Shipley, & Wachowiak, 2014), as well as in fear learning and memory (Hersman et al., 2017; Jiang et al., 2016; Unal, Pare, & Zaborszky, 2015).

Standard rodent navigation tasks do not provide fine grained behavioral information and present only limited cognitive challenge to rats and mice. While this was a constraint when animals had to be trained manually, the emergence of the rodent cognition field operating with high throughput automated assays (Brunton, Botvinick, & Brody, 2013; Dhawale et al., 2015; O'Connor et al., 2010) with psychometric training that allows controlling cognitive demand and testing well defined hypotheses will likely transform our understanding of spatial cognition. One example is the emergence and spread of rodent VR navigation systems in recent years (Aronov & Tank, 2014; Kaupert et al., 2017; Leinweber, Ward, Sobczak, Attinger, & Keller, 2017) allowing complicated navigation tasks. This also provides a segue to another important issue: mechanistic insight often emerges from probing the activity of neurons under different conditions (Sviatkó & Hangya, 2017), which type of studies have been scarce with respect to cholinergic control of spatial learning, memory and navigation. Head‐fixed VR navigation studies provide a new avenue in this direction, rendering cortical and hippocampal activity accessible for in vivo Ca‐imaging (Bittner et al., 2015; Danielson et al., 2016) and intracellular recordings (Harvey, Collman, Dombeck, & Tank, 2009). Other options include extracellular recordings, possibly combined with optogenetic tagging (Cohen, Haesler, Vong, Lowell, & Uchida, 2012; Kvitsiani et al., 2013; Lima, Hromádka, Znamenskiy, & Zador, 2009), and implantable miniscopes (Ghosh et al., 2011; Zong et al., 2017) in freely behaving rodents.

If we consider the general processing schemes of the strongly interconnected system of hippocampus, RSC, PPC, prefrontal cortex, BF and potentially other cortical and subcortical areas, it is clear that spatial cognition is likely only one aspect of more abstract processes they implement. Indeed, there is an ongoing stimulating debate whether hippocampal cognitive maps should be considered only in the spatial domain (Eichenbaum et al., 2016; Lisman et al., 2017). Additionally, it has been recently shown that RSC is involved in processing permanence and stability not only of spatial landmarks but also of actions and behaviour (Auger & Maguire, 2018). In this regard it is interesting to consider how BFCNs participate in regulating RSC functional connectivity in the default mode network (Shah et al., 2016). Therefore, research should likely be oriented towards a more general framework, putting navigation and cognitive maps into a broader perspective. This probably will require a paradigm shift that we expect will not only reveal a great deal about cholinergic modulation of spatial cognition but could also transform our understanding of neuronal information processing in general.
