# Supplementary material for: Cholinergic modulation of spatial learning, memory and navigation
Source: Eur J Neurosci. 2018 Aug 19;48(5):2199–230. doi: 10.1111/ejn.14089 (PMC6174978; doi:10.1111/ejn.14089)
Supplement: Supplementary file 1 [file EJN-48-2199-s001.docx]

Cholinergic modulation of spatial learning, memory and navigation

Hangya, Balazs; Solari, Nicola

_____________________________________________________________________________________

| Review timeline: | Submission date: 24/04/18  Editorial Decision: Minor Revision  Revision Received: 25/06/18  Accepted: 23/07/18 |  |
| --- | --- | --- |
|  |  |  |

Editor: Paul Bolam

Reviewer 1: Gordon Arbuthnott

Reviewer 2: Paul Apicella

_____________________________________________________________________________________

1st Editorial Decision	17/05/2018

_____________________________________________________________________________________

Cholinergic modulation of spatial learning, memory and navigation

Dear Balázs,

Your manuscript has been reviewed by two external reviewers as well as by the Editorial team. As you can see, the reviewers consider it a useful review dealing with a difficult field and worthy of publication in EJN.  Both, however, raise a series of points that will need to be addressed in a revised version of the manuscript.  Most of the issues that hey raise will just need clarification/expansion of the text.  As pointed out by Reviewer 2, it really would benefit from some sort of  'generalised conclusions'  and more of a critical appraisal of methods and gaps in the literature.  

We also noted the following points that need to be addressed.
- Figures need to be uploaded separately as they are currently embedded in the manuscript.
- Title page - Word and figure count needs to be added.

When revising the manuscript, please embolden or underline major changes to the text so they are easily identifiable and DO NOT leave 'track change' formatting marks in your paper.  Please ensure that you provide a text and a figure file for the Graphical Abstract (as detailed in the instructions below).  When carrying out your revisions please refer to the checklist below and visit the EJN author guidelines at www.ejneuroscience.org

When finalized, please upload your complete revised manuscript onto the website, as a Word file (.doc, or .docx).  Please also ensure that a complete set of tables and figures is included as separate files, even if these have not changed from the originals.  At this stage it is necessary to provide high resolution figures.  Please see important instructions below.

Please go into https://mc.manuscriptcentral.com/ejn - Author Centre - manuscripts with decisions where you will find a 'create a revision' link under 'actions'. We ask that you please indicate the way in which you have responded to the points raised by the Editors and Reviewers in a letter. Please upload this response letter as a separate Word (.doc or PDF) file using the file designation "Authors' Response to Reviewers" when uploading your manuscript files.  Please DO NOT submit your revised manuscript as a new one.  Also, please note that only the  Author who submitted the original version of the manuscript should submit a revised version.

If you are able to respond fully to the points raised, we would be pleased to receive a revision of your paper within 30 days.

Thank you for submitting your work to EJN.

Best wishes,

Paul & John
co-Editors in Chief, EJN


Reviews:

Reviewer: 1

Comments to the Author
For those of us still intrigued with the influence of the cholinergic systems in memory this review is a timely reminder of how little clarity has been gained from so many excellent pieces of work.  I like the complex, but probably realistic, construction of the various spatial navigational systems, though that scheme may be one of the reasons that we find the cholinergic influences so hard to understand. We could have the wrong model in the first place. Not that I have a better one, but as we get better at modelling spatial orientation in machines there may emerge a better model of how the brain predicts position and the outcome of movement within space.

However, the criticism of the methods used so far are in general fair.  In general, because I know to my cost that the p75 saponin toxin does NOT destroy cholinergic interneurons in the striatum.  We have had to wait for the ChAT-saponin to do that. Even that has problems in destroying the whole innervation.  Clearly it is time to use genetic ablation of specific receptors in specific subclasses of cells, as the basis of our pharmacological tools is eroded with more and more examples of partially specific drugs.  Even when they do better at receptor specificity their action in specific systems is hard to access.

But I digress, what is new in this review? Not so much, but it is a relatively comprehensive overview of the present state of ignorance, and so might inspire some better models or better experimental paradigms in the future.  The lack of any optimistic future plan may well be because that is another document, but it might enliven the conclusions to have a ‘cognitive map’ of future developments.
MINOR
Line 685 There are considerably "fewer" studies that "have" ... would be better
824 constraint rather than constrain
837 I'd use with rather than by

Reviewer: 2

Comments to the Author
This descriptive literature review of the role of subcortical cholinergic inputs to cortical structures involved in spatial cognition (hippocampus, posterior parietal cortex, retrosplenial cortex) attempts to shed light on the complex anatomical and functional organization of this system. Although the information provided here is certainly interesting and enlightening as regards the topic chosen, it is hard to draw up a summary and main conclusions of this review. The manuscript is relatively concise, but many parts of the text are difficult to read and a bit tedious. One reason of this difficulty is somewhat lengthy description of the research literature (e.g., pharmacological studies) without trying and drawing some generalised conclusions to help the reader in making a proper assessment of the major achievements in the reviewed field. It would have been very helpful to discuss the findings critically, identify methodological problems, and point out research gaps, rather than just summarizing the literature.

I have only a few comments to make.

I am not an expert in the field, but it seems to me that the part dealing with lesions studies (pp.13-15) does not provide a complete summary of the recent literature. The methods the authors mentioned (excitotoxic lesions) suffer from their lack of specificity. On the other hand, the reliability and efficacy of immunotoxic lesions (192 IgG saporin) is often disputed, as the authors point out. I am surprised that there is no reference to more recent methods using genetic tools for studying cholinergic neural systems. I'm not sure, but it seems strange to me that there are no attempts in this area with chemo- or optogenetic approaches.

Figure 1. I don't see where exactly is the light red structure (medial septum).

Figure 2. I don't find this figure very useful. We would have preferred to have an illustration (or panel or diagram or anything else) that helps recognize a logical way to order and link the various sections of the review.

____________________________________________________________________________________

Authors’ Response	25/06/2018

_____________________________________________________________________________________

**Response to the Editors’ comments**

*Your manuscript has been reviewed by two external reviewers as well as by the Editorial team. As you can see, the reviewers consider it a useful review dealing with a difficult field and worthy of publication in EJN. Both, however, raise a series of points that will need to be addressed in a revised version of the manuscript. Most of the issues that they raise will just need clarification/expansion of the text. As pointed out by Reviewer 2, it really would benefit from some sort of 'generalised conclusions' and more of a critical appraisal of methods and gaps in the literature.*

We thank the Editors for the positive evaluation of our manuscript. We added summaries to the sections corresponding to various methods of investigation, pointing out gaps in literature and possible future directions (Page 4, lines 123-131; page 13, lines 544-550; page 15, lines 662-663, 627-632; page 18, lines 752-757; page 20, lines 828-832). We expanded the Conclusion section as requested (Pages 21 and 22, lines 896-947, page 23, lines 965-978).

*We also noted the following points that need to be addressed.*

*- Figures need to be uploaded separately as they are currently embedded in the manuscript.*

*- Title page - Word and figure count needs to be added.*

We added word and figure count to the title page.

**Response to Reviewer #1**

*For those of us still intrigued with the influence of the cholinergic systems in memory this review is a timely reminder of how little clarity has been gained from so many excellent pieces of work. I like the complex, but probably realistic, construction of the various spatial navigational systems, though that scheme may be one of the reasons that we find the cholinergic influences so hard to understand. We could have the wrong model in the first place. Not that I have a better one, but as we get better at modelling spatial orientation in machines there may emerge a better model of how the brain predicts position and the outcome of movement within space.*

*However, the criticism of the methods used so far are in general fair. In general, because I know to my cost that the p75 saponin toxin does NOT destroy cholinergic interneurons in the striatum. We have had to wait for the ChAT-saponin to do that. Even that has problems in destroying the whole innervation. Clearly it is time to use genetic ablation of specific receptors in specific subclasses of cells, as the basis of our pharmacological tools is eroded with more and more examples of partially specific drugs. Even when they do better at receptor specificity their action in specific systems is hard to access.*

*But I digress, what is new in this review? Not so much, but it is a relatively comprehensive overview of the present state of ignorance, and so might inspire some better models or better experimental paradigms in the future. The lack of any optimistic future plan may well be because that is another document, but it might enliven the conclusions to have a ‘cognitive map’ of future developments.*

We thank the Reviewer for these deep thoughts. We expanded our Conclusion section (Pages 21 and 22, lines 896-947, page 23, lines 965-978)and the summaries of the sections corresponding to classical methods (Page 15, , lines 662-663, 627-632; page 20, lines 828-832) to suggest a brighter future.

MINOR

Line 685 There are considerably "fewer" studies that "have" ... would be better

Fixed.

824 constraint rather than constrain

Fixed.

837 I'd use with rather than by

Fixed.

**Response to Reviewer #2**

*This descriptive literature review of the role of subcortical cholinergic inputs to cortical structures involved in spatial cognition (hippocampus, posterior parietal cortex, retrosplenial cortex) attempts to shed light on the complex anatomical and functional organization of this system. Although the information provided here is certainly interesting and enlightening as regards the topic chosen, it is hard to draw up a summary and main conclusions of this review. The manuscript is relatively concise, but many parts of the text are difficult to read and a bit tedious. One reason of this difficulty is somewhat lengthy description of the research literature (e.g., pharmacological studies) without trying and drawing some generalised conclusions to help the reader in making a proper assessment of the major achievements in the reviewed field. It would have been very helpful to discuss the findings critically, identify methodological problems, and point out research gaps, rather than just summarizing the literature.*

We thank the Reviewer for the comment. We expanded our conclusions and added summaries to the sections corresponding to the different approaches to study the cholinergic system (Page 13, lines 544-550; page 15, lines 662-663, 627-632; page 18, lines 752-757; page 20, lines 828-832; pages 21 and 22, lines 896-947, page 23, lines 965-978). We hope that these additions made the manuscript easier to follow.

*I have only a few comments to make.*

*I am not an expert in the field, but it seems to me that the part dealing with lesions studies (pp.13-15) does not provide a complete summary of the recent literature. The methods the authors mentioned (excitotoxic lesions) suffer from their lack of specificity. On the other hand, the reliability and efficacy of immunotoxic lesions (192 IgG saporin) is often disputed, as the authors point out. I am surprised that there is no reference to more recent methods using genetic tools for studying cholinergic neural systems. I'm not sure, but it seems strange to me that there are no attempts in this area with chemo- or optogenetic approaches.*

Thank you for pointing this out. There is a surprisingly small number of studies that attempted optogenetic or chemogenetic manipulations of the cholinergic system, and they are not directly testing spatial navigation, rather sensory detection, discrimination, attention and fear learning. Nevertheless, we decided it is worth mentioning these studies and they are now briefly discussed in the revised manuscript (Page 15, lines 627 – 632; page 22, lines 944 - 947).

*Figure 1. I don't see where exactly is the light red structure (medial septum).*

We changed the colours so that the medial septum is easier to distinguish.

*Figure 2. I don't find this figure very useful. We would have preferred to have an illustration (or panel or diagram or anything else) that helps recognize a logical way to order and link the various sections of the review.*

We carefully considered this point. Since we could not come up with a clearer figure that better illustrates the connection between the sections, we rather attempted to improve clarity of the text by expanding session summaries (see above) and leave Figure 2 as is (except changing the colours corresponding to Figure 1), as some Readers may still find it a useful illustration of how particular functions are associated with cortical areas involved in spatial navigation.

**Response to Reviewer #3**

*This review by Solari and Hangya discusses the role of subcortical cholinergic projections in regulating various aspects of spatial learning, memory and navigation occurring in the hippocampus, as well as neocortical areas such as posterior parietal cortex (PPC) and retrosplenial cortex (RSC). While the interaction between basal forebrain nuclei (i.e. medial septum, MS) and the hippocampus in the context of spatial navigation has been studied at length, much less is known about the specific role played by the posterior basal forebrain nuclei (SI/nbM) and cortical structures (particularly PPC and RSC) in spatial cognition. This topic is addressed at some length in the review.*

*Overall the manuscript is well written and documented. It is divided mainly in two parts. The first part meticulously describes what is known about the hippocampus, PPC and RSC and their role in the various aspects of spatial navigation. The second part provides an extensive review of the role of ACh and basal cholinergic systems in modulating spatial memory. It describes studies done using ablation as well as pharmacological techniques, pointing out the importance of cholinergic signaling in navigation skills. Here, a clear tie with the initial discussion on hippocampal role in spatial navigation and the interaction between MS and hippocampus is established. Although the authors don’t come to any particularly novel conclusions, the review is thorough and balanced.*

We thank the Reviewer for the positive assessment of our review.

*The only really significant shortcoming of the review is that the second half lacks the depth of the first. In particular, it would have strengthened the review had there been an explicit discussion of how cholinergic signaling can shape the activity of these cortical regions and what role this modulation has in influencing specific aspects of behavior, so well laid out at the beginning of the manuscript.*

We now discuss how cholinergic signalling may influence different cortical regions in the context of spatial navigation by

- discussing the involvement of cholinergic cell loss in cognitive impairment (Page 4, lines 123-131; page 13, lines 544-550)
- expanding the discussion on cholinergic influence on hippocampal theta oscillation (Page 6, lines 216-219, 222-223)
- adding a new section on how cholinergic afferents may influence cortical processing in spatial learning and navigation (Pages 20 and 21, lines 834-875)

*A minor point regards the figures, especially figure 1. I understand the appeal of using 3D rendering of the brain to emphasize the spatial relation among the structures discussed here. However, this particular rendering is a little confusing.*

We changed figure 1. We hope the Reviewer will find the new version clearer.
